# Supplementary material for: Opposing patterns of abnormal D1 and D2 receptor dependent cortico-striatal plasticity explain increased risk taking in patients with DYT1 dystonia
Source: PLoS One. 2020 May 4;15(5):e0226790. doi: 10.1371/journal.pone.0226790 (PMC7197855; doi:10.1371/journal.pone.0226790)
Supplement: Attachment — Submitted filename: response to reviewers.docx [file pone.0226790.s003.docx]

***1. The finding of the study doesn’t provide any insights. Although they claim their results on simulate D2-MSNs LTP is not in line with an in vitro data, this can also due to the limitation of the model not necessarily means additional factors existed. Note that the authors didn’t fully discuss the limitation of the model.***

The computational model provides evidence for the *most* *likely* abnormality of cortico-striatal plasticity that leads to increased risk taking. Conversely, as demonstrated by our simulations, the plasticity abnormalities in a striatum with exaggerated D1- and D2-MSN LTP (as seen in the rodent model), would instead lead to risk-averse behaviour. Our model indeed reproduced the experimental data under different striatal conditions to the pattern seen in rodents; however, this reflects it being the most biologically feasible explanation for the behaviour rather than a consequence of the model’s design.

We have now included information on the above in the Discussion, and a section on the limitations of both the computational model and the *in vivo* rodent model, acknowledging these could have led to differences between computational and rodent model predictions.

On the basis of our modelling we cannot conclude that the neurophysiological properties of the rodent TOR1A model of human dystonia are compatible with risk-neutral behaviour of patients with the same TOR1A mutation. This is a significant and important insight into current computational and animal models of human disease and highlights an important area of further study.

***2. Authors seem cannot distinguish some basic ideas: cortico-striatal plasticity, synaptic strength, D1R- or D2R-expressing medium spiny neuron (or direct- and indirect-medium spiny neuron). For example, author wrote, "... post-synaptic abnormality in D2R transmission as the principle cause for ... ". There is no such receptor transmission. The idea might be dopamine transmission onto D2R-MSNs. Another example is "...D2R striato-pallidal LTP...". Authors should be aware that cortico-striatal LTP/LTD has nothing to do with the striato-pallidal and striato-nigral LTP. The cortico-striatal LTP/LTD does influence the output of D1- and D2-MSNs to GPe and SNr, but it doesn’t imply anything on striato-pallidal or -nigral LTP. Another relevant noticeable error is “… D1R-LTP…”. There is no such D1R-LTP. LTP cannot be induced on a receptor (let alone is a metabotropic receptor). I guess the authors want to express the idea: D1R-MSNs or D1-MSNs LTP. There are many more errors, which cause a huge misleading to non-field reader and research, like these above.***

We thank the reviewer for this and agree our terminology could be clearer so have revised all manuscript sections including the Abstract aimed at reducing confusion between concepts of D2R-LTP and LTP at cortico-striatal synapses at D2-MSN which are D2R dependant. We have also included a citation for Bonsi et al., 2019 to reinforce support for the idea of abnormal D2R “expression” rather than “transmission.”

***3. Citations are not appropriate. They mentioned the competition model but they didn’t cite Bariselli et al 2018 Brain Research paper, which proposed the competitive model to explain the action selection in the basal ganglia. Kravitzer et al., 2012 in the introduction cannot support RPE idea in the sentence. Frank 2005 in the introduction is also misleading. Frank did talk about how DA level affects plasticity. But, no direct statement about how positive and negative PRE shape or rely (actually are two different thing). The author also need to cite one more paper to support “rodent models” in the sentence “…proposed that patients with the TOR1A mutation should exhibit a learning strategy that is contingent with the abnormal plasticity seen in rodent models …’.***

We agree with the reviewer and have now cited Bariselli et al 2018 in the Methods, removed the Kravitz et. al., citation, and included Steinberg et. al., 2013 and Chang. et. al., 2013. We have now changed the Frank et. al., 2005 reference to Reynolds and Wickens (2002) and removed reference to “positive and negative prediction error” (see also below). We have further cited Martella et. al., 2009 and 2014 in support of the statement “…proposed that patients with the TOR1A mutation should exhibit a learning strategy that is contingent with the abnormal plasticity seen in rodent models.” We have also additionally cited Zalucusky et. al., 2016 in the discussion (as evidence in support of point 1 above) and Surmeier (2007), Albin (1989) and Delong (1990) in the introduction.

***4. The late part of the manuscript has largely focused on the idea of RPE. But, the authors didn’t mention anything in the abstract. Actually, directly use DA level concept, but not RPE, will be more related to the cortico-striatal plasticity concept. Authors try to borrow a fancy idea like RPE but fail to test it. Although phasic dopamine level change correlates with the RPE, which has been shown in many studies, a direct way to do so is to do the regression on the behavior instead of just claim RPE because of dopamine level change. Taken together, using the concept of RPE doesn’t provide extra credit to paper.***

We agree our focus on RPE signalling does not add insight into dystonia mechanisms so have modified the manuscript accordingly. Specifically, with the exception of the Methods (as the RPE signal is explicitly part of the computational model) the remainder of the manuscript has emphasised the role of phasic increases or decreases in dopamine rather than the RPE signal.
